# Making the case for workforce diversity in biomedical informatics to help achieve equity-centered care: a look at the AMIA First Look Program

**DOI:** 10.1093/jamia/ocab246

**Published:** 2021-11-22

**Authors:** Tiffani J Bright, Karmen S Williams, Sripriya Rajamani, Victoria L Tiase, Yalini Senathirajah, Courtney Hebert, Allison B McCoy

**Affiliations:** 1 Center for AI, Research, and Evaluation, IBM Watson Health, Cambridge, Massachusetts, USA; 2 Departments of Epidemiology and Biostatistics and Health Policy and Management, Graduate School of Public Health and Policy, City University of New York, New York, New York, USA; 3 Institute for Health Informatics, Office of Academic Clinical Affairs, University of Minnesota, Minneapolis, Minnesota, USA; 4 Value Institute, NewYork-Presbyterian Hospital, New York, New York, USA; 5 Department of Biomedical Informatics, University of Pittsburgh, Pittsburgh, Pennsylvania, USA; 6 Department of Biomedical Informatics, College of Medicine, The Ohio State University, Columbus, Ohio, USA; 7 Department of Biomedical Informatics, Vanderbilt University Medical Center, Nashville, Tennessee, USA

**Keywords:** medical informatics, mentoring, gender, workforce diversity

## Abstract

Developing a diverse informatics workforce broadens the research agenda and ensures the growth of innovative solutions that enable equity-centered care. The American Medical Informatics Association (AMIA) established the AMIA First Look Program in 2017 to address workforce disparities among women, including those from marginalized communities. The program exposes women to informatics, furnishes mentors, and provides career resources. In 4 years, the program has introduced 87 undergraduate women, 41% members of marginalized communities, to informatics. Participants from the 2019 and 2020 cohorts reported interest in pursuing a career in informatics increased from 57% to 86% after participation, and 86% of both years’ attendees responded that they would recommend the program to others. A June 2021 LinkedIn profile review found 50% of participants working in computer science or informatics, 4% pursuing informatics graduate degrees, and 32% having completed informatics internships, suggesting AMIA First Look has the potential to increase informatics diversity.

## INTRODUCTION 

The National Science Foundation U.S. Science and Engineering 2020 Report data showed that women accounted for about 52% of the college-educated workforce between 2013 and 2017, but only 29% of the science and engineering workforce.^1^ The under-representation of women in science, technology, engineering, and math (STEM) is also reflected in biomedical informatics; a recent study highlighted that leadership and recognition are lower for women in the field.^2^ Similarly, racial and ethnic disparities persist in the STEM workforce, with Black and Hispanic women accounting for 5% of the computing and mathematical workforce in 2019.^3^ These disparities are fueled by gender, racial, and ethnic stereotypes; isolation; lack of exposure; and lack of access to mentors, sponsors, and role models.^4^ Notably, while there continues to be abundant growth in the biomedical informatics field, a recent study of biomedical informatics doctoral programs found that Black or African American and Hispanic students accounted for ∼3% and ∼6% of biomedical informatics-related doctoral graduates from 2002 to 2017 and that in total, Alaska Native, American Indian, Black or African American, Hispanic, Native Hawaiian, and Pacific Islander students comprised 11.7% (284/2426) of all biomedical informatics-related doctoral graduates.^5^

Given the increasing influence, demand, and reliance on health information technology, it is critical to diversify the biomedical informatics workforce. Biomedical informaticians use biomedical data, information, and knowledge for scientific inquiry, problem-solving, and decision-making to improve human health.^6^^,^^7^ However, when intentionality in achieving representation and inclusivity is missing from the biomedical informatics workforce, key insights and viewpoints are also missing in the design, implementation, and evaluation of technologies. It also increases the potential for unintentional incorporation of biases into the design of technology-based interventions. Diversity leads to better problem-solving and decision-making by bringing together varying perspectives and enhancing creativity.^8^ Additionally, diversity is beneficial as salient differences may promote consideration of unique information in a group’s decision-making process.^9^ As such, building a diverse biomedical informatics workforce is necessary not only for the health of the profession,^10^ but to ensure that biomedical informatics solutions will continue transforming health and healthcare toward greater fairness and equity of outcomes by informing the development and evaluation of high-quality, culturally competent solutions, data sets, and algorithms. In short, we are all accountable for achieving rich and diverse workforces that foster inclusion and belonging as well as scientific excellence in biomedical research.^10–12^

The American Medical Informatics Association (AMIA) observed that the workforce disparities in STEM were also present throughout its organization.^13^ Addressing these disparities required an innovative solution to expose women, including those from historically marginalized communities to biomedical informatics, provide connections within the community to increase retention, and support and help advance women across their career. Although many biomedical informaticians were individually working to address the workforce gap, no comprehensive and systematic approach was in place to increase workforce diversity in AMIA. In 2017, AMIA First Look^14^ was introduced to expose undergraduate women, including women from historically marginalized communities, with an interest in STEM to the field of biomedical informatics through attendance and mentoring at AMIA’s Annual Symposium. The program, initiated by the Women in AMIA (WIA) Pathways subcommittee, was inspired from lived experiences of members and a quote by civil rights activist, founder, and president emerita of the Children’s Defense Fund, Marian Wright Edelman: “you can’t be what you can’t see.” The objective of this manuscript is to describe the AMIA First Look Program and its impact over the last 4 years on addressing workforce diversity in biomedical informatics.

## MATERIALS AND METHODS

### Program components

Over the course of developing this volunteer program, its goals have evolved to encompass the following: (1) expose women, with emphasis on those from historically marginalized communities to the field of biomedical informatics, (2) facilitate connections with women mentors in the field, and (3) equip students with resources for internship and postgraduation career “next-steps” in informatics.

AMIA First Look was launched under the umbrella of the WIA Initiative,^15^ which focuses on increasing gender diversity in the biomedical informatics field and within AMIA. Within WIA, the Pathways subcommittee works to identify opportunities to expose young women to informatics, increase the number of women in AMIA, and retain women in AMIA. Volunteers from the WIA Pathways subcommittee implemented and executed the AMIA First Look Program. The WIA Steering Committee, other WIA subcommittees, and AMIA staff and leadership provided additional feedback, mentoring, and operational support.

Each program year, Pathways subcommittee members identified public and private undergraduate colleges and universities local to the AMIA Annual Symposium (ie, within a driving distance of ∼1 h or less to Washington, DC in 2017 and 2019, and San Francisco, CA in 2018). Deliberate attention was given to include Historically Black Colleges and Universities, Tribal Colleges and Universities, and Hispanic-Serving Institutions. Subcommittee members reviewed websites for all local STEM, pre-med, pre-nursing, nursing, and allied health programs to identify a point of contact. Each program was then invited by e-mail with the AMIA First Look announcement and link to the application form. A total of 301 programs were contacted over the years with increased social media messages in 2020 with intent to increase the outreach. This invitation provided information about AMIA, program details, and participant benefits, which included complimentary conference registration, lunch for the AMIA Symposium Opening Day (2017–2019), and travel allowance ($25 for years 2017–2019). The application form required that students submit: (1) demographics, (2) undergraduate institution, and (3) informatics exposure. These recruitment activities commenced 6 months before the conference and student outreach 4 months before the conference. Since a virtual conference was held in 2020, lunch and travel allowances were omitted. However, this allowed opening the registration to programs across the nation.

Exposure to biomedical informatics was achieved by curating AMIA First Look participant access to several activities. First, participants received complimentary conference registration to the entire conference, with tailored sessions highlighted. Onsite mentors greeted participants during registration and recommended sessions, such as the Opening Plenary Session, which sought to highlight the breadth of informatics, display student research and prowess via the Student Paper Competition, or to stimulate participant curiosity about a particular topic. In 2019, subcommittee members created an Introduction to Informatics panel that included an open discussion featuring WIA members. Additionally, mentors joined the AMIA First Look Lunch, provided interactive discussions about biomedical informatics careers, gave short research talks, and accompanied participants to tutorials and scientific sessions. In 2020, AMIA First Look included a unique, game night, bonding experience with the participants called, “Informatics After Dark.” Second, participants received complimentary student membership to AMIA for 3 years with access to all online content. Lastly, participants received access to the AMIA online communities, including one created for AMIA First Look participants.

The AMIA First Look Program used different approaches to seed connections and build community for participants. First, women members of AMIA volunteered to serve as program mentors. Mentor application forms required: (1) name and organization, (2) availability for mentoring, and (3) career stage and areas of expertise. Mentors were then paired with participants ∼1 month before the conference. Second, participants were added to the AMIA First Look Connect Community,^16^ an online discussion forum with peers and mentors along with access to numerous informatics resources. Last, participants attended the Welcome Reception in the Exhibition Hall, where they spoke with representatives from academia, industry, government, and nonprofit organizations to network and guide their internship or postgraduate career next steps.

### Evaluation methods

Each year, participants were asked to complete an anonymous survey developed by the subcommittee after attending the program. Since survey questions evolved each year in response to participant feedback, data for some questions were not available for every year. Additionally, web searches from LinkedIn were performed in June 2021 to examine participants’ continued engagement with informatics from 2017 to 2020. Two authors independently reviewed each LinkedIn profile and recorded data in spreadsheet form. Data captured included graduation year, current position and organization, and internship organization. LinkedIn and survey data were analyzed across the 4 years using summary statistics including response percentages, response counts, and open-ended responses in Microsoft Excel and the result analyzer in Survey Monkey software.

## RESULTS

**Figure 1.**
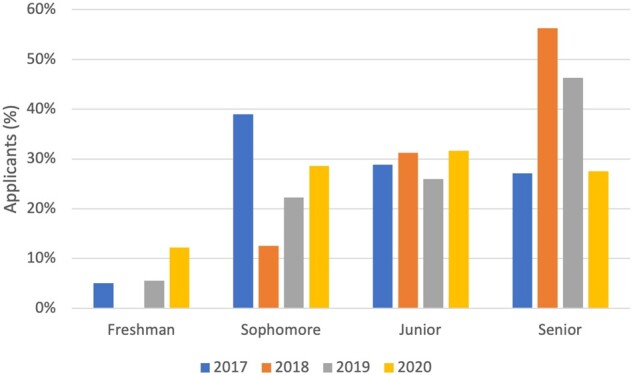
College classification of AMIA First Look applicants. AMIA: American Medical Informatics Association.

**Figure 2.**
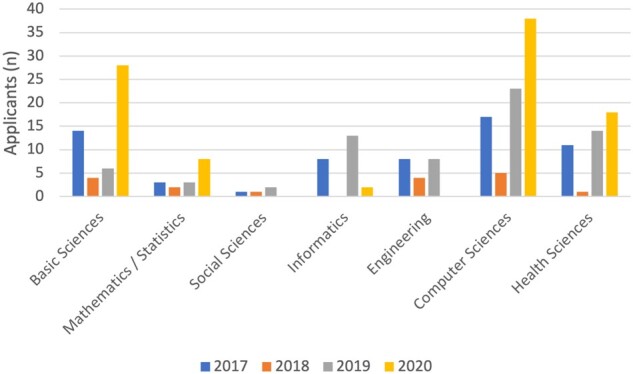
Undergraduate majors represented across AMIA First Look applicants. AMIA: American Medical Informatics Association.

**Figure 3.**
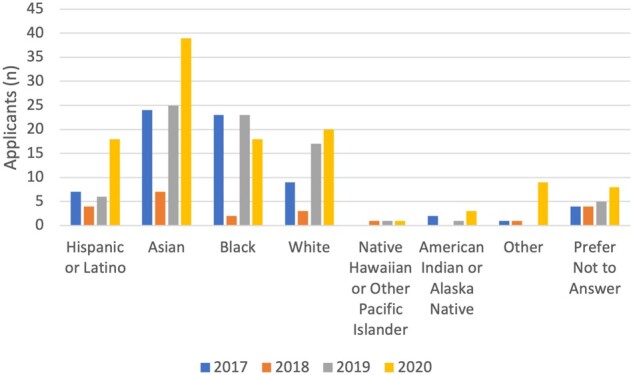
Ethnicity and socially assigned race representation of AMIA First Look applicants. AMIA: American Medical Informatics Association.

In the 4 years of the program, 227 undergraduates applied to attend AMIA First Look (59 in 2017, 16 in 2018, 54 in 2019, 98 in 2020) and 87 attended (22 in 2017, 11 in 2018, 28 in 2019, 26 in 2020); 41% (*n* = 36; 12 in 2017, 4 in 2018, 10 in 2019, 10 in 2020) were members of marginalized identity groups, Black, Indigenous, and People of Color participants, as defined by the National Institutes of Health.^17^ Participants that chose other or preferred not to answer, were not included in this grouping. Applicants were most often seniors (34%, *n* = 77), juniors (30%, *n* = 67), or sophomores (29%, *n* = 65); majors included computer science or information technology (33%, *n* = 83), health sciences (18%, *n* = 44), basic sciences (21%, *n* = 52), biomedical or health informatics (9%, *n* = 23), and engineering (8%, *n* = 20) (Figures  1 and 2). Between years 2017 and 2020, AMIA First Look was able to draw a diverse applicant pool of women who self-identified as being from the Asian (38%, *n* = 95), Black or African American (26%, *n* = 66), Hispanic/Latinx (14%, *n* = 35), American Indian (2%, *n* = 6), and Native Hawaiian (1%, *n* = 3) communities (Figure  3). Gender was included in the 2020 application with the ability to self-identify as a woman, nonbinary/third gender, transgender, and prefer not to answer. Ninety-nine percent of participants identified as female and 2% as transgender or preferred not to answer. Twenty-four participants completed an anonymous survey about their experience (9 in 2017, 1 in 2018, 7 in 2019, and 7 in 2020). Participants from the 2019 and 2020 cohorts (the first years these specific questions were asked) reported interest in pursuing a career in informatics increased from 57% to 86% after attending AMIA First Look, and 86% of both years’ attendees mentioned that they would recommend AMIA First Look to others. Participants in both 2019 and 2020 indicated that perceived barriers to careers in informatics included lack of awareness, money, and confidence.

All participants in the 2019 cohort indicated that the AMIA First Look lunch allowed them to connect with women in the field, and 90% reported that the orientation and introduction to informatics sessions were welcoming, engaging, and informational. In the Exhibition Hall, mentors guided participants around the booths, facilitating introductions to representatives while making informal introductions along the way. Importantly, several mentors and mentees reported maintaining engagement postprogram about internships and graduate school applications. In the AMIA First Look Connect Community, mentors and staff have posted resources about internship opportunities, seminars, and jobs relevant to participants. Participants expressed that meeting women experts in their desired area will continue to be most helpful. Supplementary Figures provide photographs of the AMIA First Look Program from 2017–2019. In 2020, the program was restructured to fit a virtual format, while incorporating aspects of the survey feedback, and included panels from AMIA student working groups and experts from various informatics domains, along with a unique bonding experience, “Informatics After Dark.”

Survey of participants revealed that advice and information about careers, graduate school, scholarships, and suitable programs were unmet participant needs. Networking receptions during the symposium and connections to academic partners and industry sponsors gave participants exposure to career opportunities in the field.^18–21^

The LinkedIn profile review found that 55% (40/73) of AMIA First Look participants completed their undergraduate degree program; 50% (20/40) of those were working in the computer science and data sciences (*n* = 13) or informatics field (*n* = 7). Forty-one percent (30/73) were continuing their undergraduate studies; 4% (3/73) were pursuing informatics graduate degrees (Master’s or Doctoral). Thirty-four percent (25/73) had completed or initiated at least one internship, 32% (8/25) of which were applied informatics training programs.

## DISCUSSION

It is becoming increasingly apparent that when representation is missing from the biomedical informatics workforce, key insights are omitted in the design, implementation, and evaluation of informatics-enabled solutions. However, to our knowledge, AMIA First Look is one of the only publicly announced programs specifically designed to increase diversity in the informatics field. The AMIA First Look Program approach is largely generalizable and contains components that can be reproduced in other settings to address workforce diversity.

The first reproducible component is selection of an exposure activity for students and creation of a program to serve them. Tracking participant feedback and a willingness to iterate in response is key to ensuring that the program is successful in meeting its stated goals. For example, the Introduction to Informatics session was introduced in 2019 in response to the 2018 cohort feedback that participants preferred more interactive sessions with WIA, rather than an agenda that heavily focused on attending scientific sessions. AMIA First Look also collected demographic and basic survey data on applicants and participants to better understand the impact of the program on progressing diversity and inclusion.

A second reproducible component is the creation of a community that enables belonging. In this program, we facilitated connections among fellow participants and mentors in-person as well as providing a dedicated online space for these interactions to continue. AMIA First Look has a webpage on AMIA and LinkedIn purposed to include information, opportunities, and resources for current and past participants. The Pathways subcommittee encourages continued contact between AMIA First Look mentors and participants to better meet past participant needs.

The final component is the provision of career resources to guide students in preparing for their career “next steps” after the event. We began preparing participants in the outreach letter by providing networking resources and suggestions since attending a professional conference was a new experience for many participants. Dedicating time during the conference for both formal and informal networking contributed to several opportunities for participants. Use of AMIA First Look badge labels also helped promote identification of participants, leading to additional networking within the larger community.

AMIA First Look has been a promising first step to increase workforce diversity focused on the undergraduate level, but some limitations need to be acknowledged and addressed. The low response rate (24%) to evaluation limits the iterative design of the program for a better fit. This will be addressed by soliciting assessments right at end of program day instead of emailing evaluation link few days later. Based on the STEM focused outreach, AMIA First Look attracted undergraduates who were in computer science programs (33%) and conscious efforts will be made to recruit students from other fields to draw new talent into informatics. In incorporating any of these components into their approach, other programs should note key lessons from our experience. First, administrative processes require considerable program management resources. Dedicated volunteers have led AMIA First Look to this point, but significant resources are nonetheless required to execute the program, especially when it comes to engaging participants. Second, securing financial resources for a workforce diversity program is critical. AMIA has supported this program since 2017, but sponsorships are solicited annually. Procuring additional funding would further aid program sustainability by overcoming obstacles such as travel costs—the number one reason that prevented participation. Based on continued success, AMIA First Look is scheduled to be offered in 2021. The WIA is also adopting this model to increase the number of women in the clinical informatics subspeciality.

## CONCLUSION

Women and Black or African American, Indigenous, and Hispanic/Latinx students are earning STEM degrees, but they are not represented in the biomedical informatics workforce proportionate to their overall presence in the workforce and population. AMIA First Look integrates strategies for supporting women and especially women from marginalized communities such as building an inclusive community and providing access to women mentors, both which can be significant to helping develop a sense of belonging in STEM majors and careers, overcome barriers, and persist in the STEM field.^22^^,^^23^ A biomedical informatics workforce that continues not to embrace diversity and inclusion will yield noninclusive research that stifles innovation, impedes the quality of care delivered through informatics-enabled solutions, and creates and/or widens health disparities. As diversity enhances creativity and group decision-making, it is vital to consider this as a critical component of informatics workforce development.^8^^,^^9^ AMIA First Look is one approach to addressing workforce diversity in biomedical informatics and shows promising initial results. Reproducible components of the program can inform other initiatives aiming to provide a multifaceted approach to introducing diverse undergraduate students to career pathways via a professional conference.

## FUNDING

The authors have not received direct funding to support the program and volunteer their time. The First Look Program receives support from AMIA, industry and academic partners for program logistics and operational costs.

## AUTHOR CONTRIBUTIONS

TJB conceptualized the First Look Program and led the launch in 2017 as part of the AMIA Annual Symposium; she also led the authoring and incorporated feedback from the coauthors. ABM and KSW are current Co-Chairs of the AMIA Pathways Committee and in charge of the First Look Program. ABM led the data analysis. All authors have read and approved the final manuscript.

## SUPPLEMENTARY MATERIAL

Supplementary material is available at *Journal of the American Medical Informatics Association* online.

## Supplementary Material

ocab246_Supplementary_Data
