# Supplementary material for: Making the case for workforce diversity in biomedical informatics to help achieve equity-centered care: a look at the AMIA First Look Program
Source: J Am Med Inform Assoc. 2021 Nov 22;29(1):171–5. doi: 10.1093/jamia/ocab246 (PMC8714276; doi:10.1093/jamia/ocab246)
Supplement: ocab246_Supplementary_Data [file ocab246_supplementary_data.zip › ocab246-suppl_data/Appendix.docx]

Appendix: Photographs of the First Look Program (2017-2020)

**Goal 1: Expose women to the field of biomedical informatics.**

Figure 1a: 2017 AMIA First Look students leading the discussion during the W14: Enabling Innovation and Collaboration in Informatics and Health Care Research Collaborative Workshop

Figure 1b: 2018 AMIA First Look students engaging with Dr. Adriana Arcia during the W27 Patient and Consumer Engagement in Health Information Technologies Collaborative Workshop

**Goal 2: Facilitate connections with women in the field.**

Figure 2a: 2018 AMIA First Look student and mentor (Dr. Jessie Tenenbaum and Bria Massey)

Figure 2b: 2019 AMIA First Look students and mentors post-networking lunch

**Goal 3: Equip students with resources for internship and post-graduation career “next-steps” in informatics.**

Figure 3a: 2018 AMIA First Look students networking with program sponsor

Figure 3b: 2019 AMIA First Look student speaking with exhibitors to learn about internships, graduate schools, and career opportunities
